# Perspectives of older adults with a chronic condition on functioning, social participation and health: a qualitative study

**DOI:** 10.1186/s12877-021-02365-w

**Published:** 2021-07-09

**Authors:** Leen De Coninck, Anja Declercq, Leen Bouckaert, Mieke Vermandere, Maud J. L. Graff, Bert Aertgeert

**Affiliations:** 1 Department of Public Health and Primary Care, KU Leuven, Kapucijnenvoer 33, blok J, PB 7001, 3000 Leuven, Belgium; 2 CEBAM Belgian Centre for Evidence-based Medicine vzw, Kapucijnenvoer 33, blok J, PB 7001, 3000 Leuven, Belgium; 3 Department of Occupational Therapy, Artevelde University of Applied Sciences, Voetweg 66, 9000 Ghent, Belgium; 4 LUCAS Centre for Care Research and Consultancy & CESO Centre for Sociological Research, KU Leuven, Minderbroedersstraat 8, PB 5310, Leuven, Belgium; 5 Scientific Institute for Quality of Health Care and Department of Rehabilitation, Donders Center for Cognition, Brain and Behavior, Radboud University Medical Centre, Houtlaan 4, 6525 XZ Nijmegen, the Netherlands

**Keywords:** Older adults, Functioning, Social participation, Adherence, Person-centered care, Occupational therapy

## Abstract

**Background:**

Problems with mobility, functioning and social participation make living independently difficult for frail older adults. To continue living independently, therapy adherence is a prerequisite. The causes for non-adherence among older adults are multiple and complex, which is why insight into older adults’ perspectives regarding their functioning is an essential factor to increase therapy adherence.

This study investigates the perspectives of older adults on their functioning, social participation and health, and the factors influencing these elements.

**Methods:**

We conducted a qualitative study on the older adult’s perceived functioning, social participation and health. Fourteen home-dwelling older adults suffering from chronic health issues were purposively selected.

Semi-structured interviews were conducted with open-ended questions.

Data were analysed following the Basic Logical Model of Abduction and Creswell’s coding method.

**Results:**

Assistive devices, the older adult’s dwelling and living environment, professional and informal support, and medication are perceived as important determinants for retaining functioning and social participation.

Attitude, social influence and personal effectiveness were found to influence whether a person performs or participates in an activity. A person’s attitude is related to the significance the activity has to that person, the activity’s importance, personal wellbeing, the person’s values, and their desire for autonomy. Peers and children have a social influence on the level of activity of the older person. Traditions, in particular religious activities, along with personal effectiveness are motivating factors determining whether a person performs or participates in an activity. Personal effectiveness is linked to the person’s belief in their personal competencies and to the relationship between effort and result.

Finally, it appears that the type of coping strategy the older adult adopts, has an influence on their behavior. The participating older adults often used remarkable problem-focused strategies, which had a positive effect on their level of autonomy.

**Conclusions:**

Older adults have identified barriers and facilitators that influence their level of functioning and social participation. These findings help to create a framework for maintaining and increasing therapy adherence, which may be helpful in facilitating occupational therapists and other healthcare professionals to detect determinants of therapy adherence.

**Supplementary Information:**

The online version contains supplementary material available at 10.1186/s12877-021-02365-w.

## Background

Physically frail older adults experience problems with mobility, functioning and social participation. Despite these problems, they want to live at home for as long as possible [1–3]. Participation in activities and in social life, as well as an adapted living space are important for people to be able to stay in their homes for as long as possible [4–6]. The use of home modifications results in improved function and decreased falls which can be achieved with the help of occupational therapists, whose contribution is often vital [6]. Therapy adherence, including adherence in performing home modifications and using assistive devices is a prerequisite for success.

However, lack of therapy adherence is a common problem among older adults with chronic conditions [7, 8]. There are various, interplaying reasons for non-adherence [9, 10]. Person-related factors play a role, such as the degree to which an activity is tailor-made. Interventions regarding age-related issues, such as the risk of falling, need to address the clients’ attitudes and beliefs, as well as their physical capacity. Insight into older adults’ perceptions regarding their functioning and social participation is a precondition for person-centered advice and for successful treatment that strives towards behavioral change [11–14].

Therefore, healthcare professionals such as occupational therapists inform older adults on their health. They strengthen the older adult’s motivation and competence to make health-related decisions by increasing health literacy. This extended knowledge increases the older adult’s personal empowerment and autonomy when carrying out activities at home or partaking in social and cultural events [15].

To pursue successful and sustainable treatment, behavioral change of the older adult is necessary. The TransTheoretical Model of Change (TTM) describes how a sustainable change in behavior can be obtained by following a well-structured procedure. TTM consists of five stages, i.e., pre-contemplation, contemplation, preparation, action and maintenance. The approach should be adapted to the level of function the patient is at [16, 17].

**Fig. 1:**
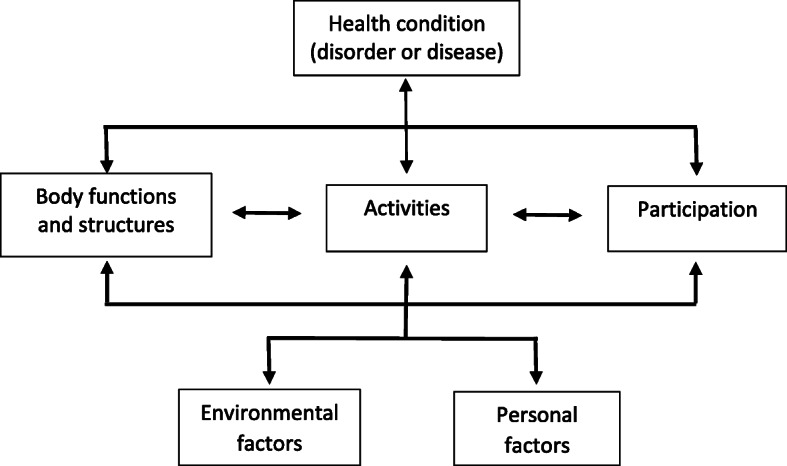
ICF-model [20]

Occupational therapists’ work is evidence- and occupation-based. Early pioneers in occupational therapy conceptualize ‘occupation’ as an active participation in self-maintenance, work, leisure, play and rest [18]. Performing occupation-based interventions means that the occupational therapist develops a holistic view of the client by looking at all the client’s components as described in the International Classification of Functioning, disability and health framework (ICF-framework) and determining how they relate to each other. The components of the ICF-framework are as follows: body functions and structures, activities, participation, as well as environmental and personal factors [19, 20] (Fig. 1).

A step-by-step treatment plan to realize a high-quality occupational therapy intervention to modify the home consists of assessing a person’s abilities and their home environment, developing occupational goals, drawing up and implementing an intervention plan to overcome barriers, and training the older person and/or their informal caregiver(s) to complete daily activities using environmental support [6, 21]. Stark [21] developed a list of 16 personal factors and conditions that expert occupational therapists routinely assess and integrate in their home modification intervention plans, among which the housing type, the ability of the older adult to care for and maintain the home modifications, their personal assistance preferences, their physical assistance available, their readiness for change and the ability to manage their finances. These factors are based on data from interviews with and observations of occupational therapists. However, Stark did not include views of the older adults themselves in her study. To increase the treatment effect, there is a need for complementary factors that considers the older adult’s perspective regarding their functioning, social participation and health. The present study adds to Stark’s model by also including the perspectives of older adults.

## Objective

This study investigates the perspectives of home-dwelling older adults on their functioning, social participation and health, and on the factors influencing these capacities and their health.

## Methods

### Design

In-depth interviews with home-dwelling older adults were performed, using open-ended questions about their perceptions of their functioning, social participation and health, and the factors that have an influence on these elements. The theoretical framework underpinning this study is the Basic Logical Model of Abduction which uses abductive analysis as its qualitative data analysis approach [22]. Abductive analysis aims at generating novel theoretical insights through a dialogue between sensitivity for cultivated theory and methodological heuristics [23]. In this way, the Basic Logical Model of Abduction emphasizes that research can be both inductive and deductive. Abductive analysis allows researchers to let themes, patterns and categories emerge from data (inductive) and, on the other hand, rely on existing analytical categories obtained from previous theories (deductive). Insightfully abducting during analysis consists of developing themes, codes and categories that structure data. When organizing and structuring the analyzed information, it might turn out that it fits within existing models. These existing models provide insights which, in turn, lead to further analysis, so that broader and more extensive knowledge is gained. Abduction challenges researchers to develop their theoretical repertoires throughout the research process rather than setting all preconceived theoretical ideas aside during the research project [23–25].

### Sample selection and participants

Older adults suffering from chronic health issues were identified using the networks of the primary healthcare professionals involved in this study. Respondents were informed by phone about the project and what would be expected from them. If they agreed to participate, an appointment was made for a face-to-face interview in their home. All participants gave their informed consent.

The sample consisted of people aged 65 or older who were suffering from chronic health issues, home-dwelling (alone or otherwise), and able to communicate in Dutch.

People diagnosed with dementia, irreversibly bedridden, or requiring palliative care were excluded.

Convenience sampling was used, as we opted to strive for the greatest possible diversity and not for saturation. We did, however, set a minimum of 12 interviews. To capture major variations, we varied extremes in terms of age, level of mobility, residential situation and living situation. Because the initial selection did not provide sufficient information, we included two extra respondents. One of the additional respondents lived in an assisted environment and the other one had to deal with an external inhibiting factor, the burden of a disabled child (Table 1).

**Table 1:** Demographic characteristics

Respondent	Gender	Age	Marital status	Reported health problems	Level of mobility	Residential situation	Living situation
1	female	80’s-90’s	widow	blood pressure, cardiac	restrictions on moving in- and outdoors	townhouse with stairs	living alone
2	male	80’s-90’s	widower	balance, vision	restrictions on moving in- and outdoors	townhouse with stairs	living alone
3	male	80’s-90’s	widower	memory	no restrictions on moving indoors; partial restrictions on moving outdoors	townhouse with stairs	living alone
4	female	100’s-110’s	widow	vision, falls, arthrosis	restrictions on moving in- and outdoors	townhouse with stairs	living alone (week); living with children (WE)
5	male	70’s-80’s	married	hernia	No restrictions on moving indoors; partial restrictions on moving outdoors	flat with elevator	living with wife
6	female	80’s-90’s	widow	normal aging diseases	no restrictions on moving indoors; no restrictions on moving outdoors	flat with elevator	living with partner
7	male	90’s-100’s	married	sleep, presbyacousis, mobility	no restrictions on moving indoors; partial restrictions on moving outdoors	detached house with stairs	living with wife
8	male	90’s-100’s	married	vision (blind), arthrosis, mobility	restrictions on moving in- and outdoors	ground floor of townhouse	living with wife
9	female	70’s-80’s	widow	mobility	restrictions on moving in- and outdoors	townhouse with stairs/stairlift	living alone
10	female	60’s-70’s	widow	mobility, backache	restrictions on moving in- and outdoors	flat with elevator	living alone
11	female	80’s-90’s	widow	mobility, intestinal	No restrictions on moving indoors; partial restrictions on moving outdoors	townhouse with stairs	living alone
12	female	70’s-80’s	married	rheumatoid arthritis, osteoporosis, dented vertebra	restrictions on moving in- and outdoors	detached house with stairs/stairlift	living with husband
13	female	60’s-70’s	married	arthrosis, heart rhythm, glaucoma	no restrictions on moving in- and outdoors	detached house with stairs	living with husband and sun with special needs
14	male	70’s-80’s	married	arthrosis, glaucoma	no restrictions on moving indoors; partial restrictions on moving outdoors	Assisted flat with elevator	living alone, separately from his wife

### Data collection

An interview guide was developed based on the components of the Canadian Model of Occupational Performance and Engagement and on the Canadian Occupational Performance Measure [26, 27] (Additional file 1: Interview Guide).

The interviews lasted 30 min on average, were audiotaped and transcribed verbatim.

The questionnaire was pilot-tested. Based on these pilot interviews, adjustments were made to seek more meaningful answers. For instance, the question *‘What facilitates you in doing these activities/in participating in these activities?’* was supplemented with in-depth questions that inquired about facilitating attitudinal aspects, mental capacities, physical capacities and environmental influences.

### Data analysis

The researcher performing the interviews also transcribed the audiotapes. The interviews were then analyzed by two people independently of one another, without using any software.

Data were analyzed following Cresswell’s methodology [28]. Creswell describes a systematic process for data encoding, during which statements are analyzed and categorized into meaningful clusters representing the investigated phenomenon. The different steps in the analysis process are data management, reading and taking notes, describing, classifying and interpreting, reporting, and visualizing.

Two researchers independently analyzed the text, made notes (short sentences, ideas or core concepts) and attributed initial codes to two of the interviews. The findings were discussed in detail to reach agreement on how to further analyze the other interviews. The researchers divided the data into themes and text was grouped under meaningful categories. One researcher analyzed the remaining 12 interviews based on the agreement that emerged from the discussion. The second researcher reviewed this analysis and discussed alternatives with the first researcher. If discussions did not end in consensus, a third researcher was consulted. In the final phase of the cycle, the essence was captured in writing.

**Fig. 2:**
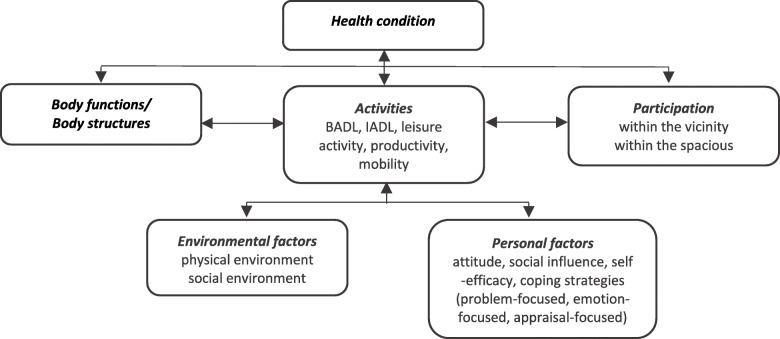
Analysis framework, based on ICF, CMOP-e and ASE-model

During the analysis process, subthemes were derived from the data using abductive reasoning. In the course of subsequent analysis, we gradually discovered theoretical frameworks to operationalize concepts from the ICF, the Canadian Model of Occupational Performance (CMOP-e) of the Canadian Association of Occupational Therapists [29, 30], de Vries’s Attitude-Social Influence - Self-Efficacy Model (ASE-model) [31] and Moos’s coping strategies [32] (Fig. 2).

In the final step, the results were contextualized. When the results were reported, a distinction was made between the descriptive display of what the respondent indicated on the one hand, and the direct representation of the respondent’s perception concerning functioning and determinants of behavior, changes in behavior and coping strategies on the other hand.

### Reflection on the role of the researchers

The research group consisted of a multidisciplinary team, knowledgeable in the fields of sociology of health, occupational therapy, gerontology and family medicine. As a result, data analysis was influenced by these various academic and professional backgrounds.

## Results

### Demographic characteristics

Fourteen older adults were interviewed (eight women and six men), with their ages ranging from 65 to 101 years. Because we strived for maximum diversity, we included one respondent who was slightly younger (a difference of less than 1 year), but who had severe mobility problems. This person met all the other selection criteria. All participants indicated they had chronic health problems which impacted their functioning. The number of indicated problems varied from one to three (Table 1).

The analysis of the older adult’s experiences is grouped under three themes: the older adult’s perceived level of activity and participation, their perceived health, and the factors they perceive as influencing their activity level and participation.

### Perceived level of activity and participation

The ‘activity and participation’ theme was categorized into five subthemes: Basic Activities of Daily Living (BADL), Instrumental Activities of Daily Living (IADL), productivity, leisure activity and mobility.

Eleven respondents indicated being able to handle BADL on their own. Three respondents indicated requiring assistance with dressing and undressing, with the extent of the required help varying from completely dependent to only needing help closing small zippers. About half of the respondents (eight) stated having problems with climbing stairs.

All respondents indicated perceiving limitations regarding IADL, productivity, leisure activity and mobility. Nearly all respondents reported being able to prepare small snacks. Doing laundry, cleaning, running errands and working in the garden were most problematic. The three respondents who reported problems with BADL reported performing only a few IADL activities. These respondents were over 90 years of age and two of them had a profound visual impairment. The remaining limitations were mostly related to strength, flexibility, endurance and balance. Strength refers to a global reduction in strength as well as specific strength loss in the hand region. The reduction of flexibility was mostly at the level of the hands and wrists. Visual impairments greatly limited being active in the field of IADL.

*“In the kitchen, it’s my wife who does everything. I can’t do anything because I can’t see it. I can’t feed the pets anymore either, my wife does that. My wife also does the garden. When I go outside I take my walking-frame, but last Sunday I took my cane. I should have used my walking frame...” (90’s-100’s - man with visual impairment and mobility issues)*

One respondent had a part-time paid job. Two respondents gave informal care or did chores for family members and friends, which varied from having a fixed schedule to sporadic services. One respondent wanted to be more productive, but saw no opportunities.

All respondents (*n* = 14) reported that the variety of pastimes available to them had been reduced, ranging from a slight to a great extent. The decrease included both outdoor activities and individual indoor activities, such as not being able to read a book.

All respondents reported a decrease in mobility, with six of the respondents using an ambulatory aid. One of these respondents indicated having very limited mobility due to multiple physical limitations, including severe visual impairment. This person no longer left the home to participate in social activities, but often received visits at home. All respondents had a decreased ability to travel long distances. Two respondents communicated being able to ride a bicycle but only a bicycle adjusted to their needs. Five respondents still drove a car. Three respondents still went on holidays, of which one respondent still traveled like he used to in the past. The second respondent went on large trips, but only with a group. The third respondent indicated only travelling in comfortable circumstances. For example, he preferred to pay more for comfortable transportation. Overall, the respondents frequently relied on assistive devices or opted for alternative activities, both inside and outside the home. Nevertheless, the use of creative and less creative solutions did not remove all mobility restrictions, which led to less or adapted participation in previously familiar outside activities.

*‘The large walks have been left behind. Up until about four years ago, those still went fine, despite my back issues. And cycling, mostly uphill and downhill, I don’t like going straight ahead all the time. It seems like it’s more exhausting if I don’t have variation.’ (70’s-80’s - man with lower back-pain)*

### Perceived health

Most of the older adults (*n* = 11) indicated being in good health, independent of the number of health issues they had, and the degree of limitation they experienced. Three people rated their health more poorly, of which two had severe mobility limitations. Both respondents wanted to stay active, despite their limitations. Nobody said their health was bad.

*‘I’m generally good, it’s just my walking that’s less good, but otherwise I’m healthy. My health never deteriorates. I’m happy with what I can still do.’ (70’s-80’s - woman using a walking aid)*

### Factors older adults perceive as influencing their activity level and their participation

Categories that are mentioned as extrinsic factors that influence functioning are assistive devices, the dwelling and living environment, professional and informal support and medication. These factors can be both facilitating and inhibiting.

The most used *assistive devices* pertained to mobility (*n* = 8). Five respondents go outside with a walker or a rollator. One respondent utilized a cane outdoors. Two older adults used a stair lift. Three older adults were advised to use a cane but refused to do so because of perceived stigma. The respondents stated that aids can facilitate one aspect of functioning, but also bring about new limitations in other areas. For example, a walking aid facilitates walking, but causes issues when trying to transport things or perform certain other actions while walking. Kitchen appliances are less often used (*n* = 2). Sometimes, specific kitchen appliances were used for household tasks that require hand-strength, such as a bottle-opener with an elongated handle.

*‘My walker and my rollator, these are my freedom. I use my walker upstairs and it stays there. I have my rollator downstairs. I always take my rollator with me when I go outside.’ (70’s-80’s - woman living alone)*

All respondents reported that their *dwelling and living environments* were adapted for them to function optimally. In two of the nine houses with more than one floor, a stair lift was installed. Two other older adults did not use the upper floors anymore but did not find their home not adapted to their needs. All older adults who lived in an apartment made use of the elevator present in the building. One respondent recently moved to assisted living and said this facilitated mobility. The majority of the older adults indicated that the neighborhood was not sufficiently adjusted for people to move around safely. Stairs, the type of terrain and support points in the neighborhood were indicated as important determinants for mobility.

The help from *professional services* was perceived as facilitating, albeit with certain limitations related to frequency and time. For example, certain types of services were not provided on weekends. The professional services consisted of help at home or respite care. Examples of services at home are home library service, garden service, meals on wheels and home care. Examples of respite care are day-care centers and a short stay at a care facility. The respondents generally perceived *informal assistance* by family and neighbors as facilitating. The respondents also mentioned disadvantages. Informal caregivers, just like professionals, are not always available. Informal caregivers have their own needs and worries and that can interfere with the provided informal care.

*‘Especially strength in my hands, that’s what I experience. When I need to turn the key, then I need to do that twice and twist, or if I need to open a jar, then I give it to the boy next door.’ (80’s-90’s - woman living alone)*

Taking *medication* is perceived as normal. Still, older adults indicate that medication is not enough to alleviate all pain or discomforts, for example rheumatic pains. Despite optimal medication-intake, they still experience limitations. They also indicate that for certain afflictions, no suitable medication solutions are available yet.

### Perspectives on determinants of functioning, social participation and health

Characteristics of attitude, social influence and self-effectiveness, as well as the applied coping strategy determine whether or not a person performs or participates in an activity.

### Attitude

The older adult’s attitude towards their – possibly reduced – functioning was determined by one or more of the following categories: significance, necessity, wellbeing, belief and desire for autonomy.

The care for a pet, partner, friend, grandchild and their property, as well as self-care were meaningful actions that motivated the older adult to remain active. Things they did not perceive as meaningful did not motivate them to be active or to participate socially.

*‘ … I have trouble talking to other people, having a conversation, that’s just not me … my mother was like that as well. Everyone knew she didn’t do chatting.’ (100’s-110’s - woman living alone)*

Activities that are normally not performed by older adults because of bodily limitations were still performed in extraordinary situations. Enjoying the things they do was often mentioned in relation to ‘doing things together’ and doing things that lie within their sphere of interests, like hobbies. Both increased their feeling of wellbeing.

Being convinced that a certain way of behaving contributed to better physical or mental health, or a certain mental attitude can lead to being active or to healthier active behavior.

*‘I often tell myself “exercise in the morning, every day”. But some do nothing, I wonder how people with back pain can sit for hours without adopting an ergonomic posture.’ (70’s-80’s - cohabitating man)*

The desire for autonomy was the greatest motivator for staying active. All older adults expressed the wish to maximally maintain their privacy.

*‘Get up, washing my privates and my face, I’ve already washed my privates when the nurses come to shower me. I want to wash my privates myself.’ (100’s-110’s - woman living alone)*

### Social influence

The perceived social pressure and support that motivated being active or that influenced the frequency of participation mainly consisted of the non-professional environment, more specifically the children and peers (friends and acquaintances). The most important peer in this matter was the partner. Perceived social pressure and support was seldom associated with the professional environment.

Traditions and religious activities were events that the older adults experienced as manifestations of social norms and as incentives for remaining active. Weekly church services and leaving the house for family events were the most common examples. The importance the older adult assigned to their (self-) image was a self-enforced norm, but it was often strengthened by a societal norm.

*‘I refuse to wear a hearing aid. I think it is a sign of old age …*. *My wife does everything in our household. Every two weeks the cleaner comes. Everything else we do ourselves … I don’t like the idea that someone would think, I’m here with pensioners who neglects everything around them … ’ (90’s-100’s - cohabitating man)*

The older adults only mirrored active behavior of active peers in their immediate environment, since these tend to have a motivating influence on them.

*‘I think it’s because you’re still in your regular environment. You see people doing things and think “I can do that too” and you do it too. Because if I were to end up in a nursing home, it would be over quickly.’ (80’s-90’s - cohabitating women)*

### Self-efficacy

The older adult’s belief in their self-effectiveness was a determining factor for whether they attended activities and participated. The decision to be active was determined by the balance between the effort put in and the result this effort provided. Most older adults could estimate how much effort they needed to complete a certain action and how long they could maintain this.

*‘Running I’ve thought of that, but why should I do that? I can walk just as well.’ (70’s-80’s - man living alone)*

Eleven respondents adjusted their actions according to their insight into their own effectivity, but this was not the case for all older adults. Three older adults exceeded the limits of their comfort zone to still be able to perform certain actions.

*‘When I wake up, I think “I hope I don’t have pain today for a change”. Rheumatism, that sometimes takes two hours. And I think “If I didn’t experience pain, I might miss it” … The laundry does not go as smoothly as it used to, where it used to take an hour, I now take one hour and a half. It’s the same with cleaning.’ (70’s-80’s - woman living alone)*

### Coping strategies

The older adults used various coping strategies, with problem-focused strategies being the most frequent and emotion-focused and appraisal-focused strategies being less frequent.

Older adults who employ *problem-oriented coping* strategies had a positive attitude and were more determined, since they had learned to solve problems on their own over the course of their life. To keep stress under control, these older adults dealt with problems actively. They looked for ergonomic solutions like sitting down whilst ironing, performing an action in steps, purchasing multiple walking aids to utilize in various locations, or they purchased ergonomic products.

*‘My husband and I regularly go cycling with our son. Walking long distances is no longer possible because of my foot injury. Cycling keeps us moving.’ (60’s-70’s - woman living with sun with special needs)*

Looking for social support from a partner, the neighbors or someone from the wider environment, such as the cashier, was often employed. Aside from that, older adults would anticipate possible issues like back pain by staying within their limits or working more slowly.

An *emotion-focused coping* strategy that was employed was seeking diversion, such as thinking of the grandchildren and meditation, by reading a prayer. Emotion-focused coping involves managing the emotional distress that is associated with a problematic situation [33]. It includes all the regulative efforts to diminish the emotional consequences of stressful events [34].

*‘When I am in a lot of pain, I pray or think about my grandchildren. It distracts me from the pain.’ (60’s-70’s - woman living alone)*

An *appraisal-focused coping* strategy that came to the fore was humor, serving as a comforting thought. Appraisal-focused coping refers to the way a person thinks about the stressors or circumstances they are confronted with. People may alter the way they think about a problem by adapting their goals and values, such as by finding humor in a situation [33].

*‘Putting things into perspective through humor … we try and agree with each other as much as possible. What’s changed is that instead of softly whispering to each other we now have to yell at each other.’ (90’s-100’s - cohabitating man)*

## Discussion and conclusion

### Overall findings

Our study sheds light on the themes that need to be mapped to increase physically vulnerable older adults’ therapy adherence.

Assistive devices, the older adult’s dwelling and living environment, professional and informal support, and medication are perceived determinants of functioning, social participation and health that are important. These determinants can be both facilitating and inhibiting. This knowledge confirms the importance of Starks’ findings that occupational therapists inquire about the presence of physical assistance, housing type, structural conditions of the home and the available space and layout. This information is gathered to support clinical reasoning. Also, questions are asked about the person’s preferences for social support and personal assistance [6, 21]. Considering this information will increase adherence to the occupational therapy recommendations. A study on beliefs of people living with rheumatoid arthritis showed that levels of concern about side effects of medication were high and related to non-adherence [35].

Our study also indicates that the influence of medication is a factor that must be considered even more to increase adherence for recommendations. Thus, our findings are both consistent and complementary with the literature on influence of professional and informal support on the level of activity and participation, and on therapy adherence [6–8, 14, 21, 35, 36].

Attitude, social influence and personal effectiveness were found to be of influence on the level of activity and social participation. A person’s attitude is related to the significance the activity has for that person, the activity’s importance, personal wellbeing, conviction and the desire for autonomy. Peers and children also have a social influence on the older person’s activity level. Traditions, and religious activities in particular, along with personal effectiveness are motivating factors determining whether to perform or participate in an activity. Palominos’ study on persons with rheumatoid arthritis states that a patient’s beliefs influence the global impact on therapy adherence and coping patterns. The study proved that the older adult’s belief that they were not able to control pain and the course of disease, has a statistically significant correlation with disability. Personal effectiveness is linked to the older adult’s belief in their personal competencies and to the relationship between effort and result [35]. Recent work also demonstrated that the older adults’ sense of confidence in using assistive devices drives continued participation in a specific activity [8, 37]. This is in line with our study indicating that attitude, social influence and personal effectiveness influence on the level of activity and social participation of the older adult. In addition, our study operationalizes these factors from the perspective of the older adult, which can facilitate occupational therapists and other healthcare providers to work person-centered. Preferences regarding personal assistance, readiness for change, adherence and concern for aesthetics are personal factors considered by occupational therapists to inform their clinical reasoning [21]. Supplementing these factors with gaining insight in the older persons’ attitude, their sensitivity to social influence and their way they perceive their personal effectiveness completes the relevant information which allow occupational therapists to deliver complex interventions with high fidelity.

Finally, it appeared that the type of coping strategy the older adult adopts has an influence on their behavior. In our study, the older adults often used remarkable problem-focused strategies, which had a positive effect on their activity level and participation. Our findings are consistent with those from previous research into other health behaviors which generally show an influence of coping on preventive behavior and wellbeing [38, 39].

The use of a person-centered approach in care for older persons focuses on the older adult as a person with the ability to make autonomous decisions. Incorporating the uniqueness of each client’s situation into the daily routines of the health professional, leads to increased well-being and satisfaction [40]. Well-being and satisfaction promote improved adherence and self-management behaviors in people with chronic health issues [41–43]. Therefore, it is necessary for a health professional to have insight in the client’s perspectives. Interaction with frail older adults and their informal caregivers must be part of a geriatric comprehensive assessment to define the impact of illness and symptoms on a daily-to-day life [44].

### Conceptual framework

**Fig. 3:**
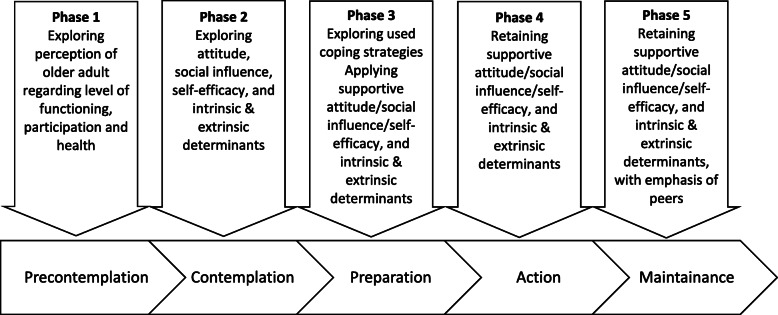
Conceptual model to determine factors influencing functionality, social participation and health

Therapy adherence is complex and has different determinants. The link between these determinants and therapy adherence is the older person’s personal perspective on it. On the basis of the present study’s findings, a conceptual framework based on TTM has been developed that might serve as a helpful tool to take into consideration the older adult’s perspective on their functioning, social participation and health (Fig. 3).

In our study, the older adults indicated being generally happy with their situation, independently of their level of functioning. ‘Generally happy with the functioning level’ and ‘the experience of being in good health’ are positive mindsets in the frame of perceived life quality, but not in the frame of therapy adherence, as these older adults show no interest in the benefits of a higher level of functioning. Within the TTM, these older adults are situated in the pre-contemplation phase. To take them to a higher level, it is desirable to have a detailed knowledge of their level of functioning, social participation and health (Fig. 3 phase 1). Subsequently, the healthcare professional must have an overview of the facilitating and limiting intrinsic and extrinsic factors that influence the older adult’s functioning (Fig. 3 phase 2). The factors that facilitate behavioral changes should dominate the limiting factors. For instance, ‘wanting to mean something to others’ could be the primary motivation to remain active despite physical limitations. Previous research shows that these factors must be incorporated in the goalsetting to improve the outcome of an intervention [45].

The older adult who is already aware of their limitations is situated in the contemplation phase or in a higher phase. These people can be gradually informed with regard to possible consequences and a suitable approach to their limitations. A gradual approach is essential to avoid forcing or provoking the older adult, as that might lead to resistance. The intrinsic and extrinsic factors (Fig. 3 phase 3) play a key role in whether the change from intention (preparatory phase) to behavior (action phase) takes place, i.e., therapy adherence. Therefore, facilitating factors need to be optimally utilized and inhibiting factors need to be identified to be neutralized early in the process or, if possible, turned into facilitating factors.

The intention to achieve behavior and behavioral changes (contemplation phase) is jointly decided by the attitude, social norm and own effectivity. From the data-analysis we can deduce that the attitude of the older adult towards their -whether or not reduced- functioning and social participation is determined by the action’s meaningfulness and necessity, as well as the older adult’s wellbeing, belief and desire for autonomy. De Vries [31] claims that social influence can be categorized into three factors: the perceived social pressure, the social norm and modelling. Having insight in these factors helps healthcare workers to adequately prepare the older adult for making effective behavioral changes (Fig. 3 phase 4). This process is facilitated by means of an overview of the coping strategies that the older adult employs. Using the environment, such as peers or informal caregivers who support the older adult, can help to give the new behavior a sustainable place in the older adult’s life (consolidation phase) (Fig. 3 phase 5).

The findings of our study also align with the models of health literacy. Health literacy entails people’s knowledge, motivation and competences to obtain, understand, appraise, and apply health information in order to make judgments and take decisions in everyday life to maintain or improve their quality of life throughout their life [15]. Our conceptual framework serves as an asset for strengthening the older adult’s empowerment and supports the healthcare professional in coaching the older adult towards the last step of health literacy, i.e., applying health information to make decisions to maintain and improve their health.

Using the conceptual model leads to a more detailed overview of and better insight into the various determinants that influence the older adult’s functioning, social participation and health. The developed conceptual framework can be a valuable support to the occupational therapist and other caregivers who work closely with vulnerable older adults to encourage better therapy adherence.

### Strengths and limitations of this study

This qualitative study gives insight into the factors that play a role in the functioning and participation levels of home dwelling older adults. Qualitative research allowed to obtain in-depth insights in the perspectives of this population. In this way, factors that need to be considered to pursue client-centered successful treatment of vulnerable older adults were identified. Follow-up quantitative research is necessary to confirm the outcomes.

A limitation to the study is the number of respondents. Although we strived for maximum diversity in our sample, it is still possible that certain factors were missed.

Whether or not to use software for qualitative data analysis can be another point of discussion. Qualitative Data Analysis Software (QDAS) applications are designed for qualitative research assist in the management and analysis of qualitative data. Preparing, organizing and managing data are facilitated by using QDAS. QDAS applications however do not define conceptual categories or themes, develop conceptual diagrams, write memos and journals, gain insight into phenomena, or develop theoretical understanding [46]. Since we included a limited number of respondents (*n* = 14), preparing, organizing and managing data was not an insurmountable work. Therefore, no software was used.

The conceptual framework can be used to identify the factors that determine the level of functioning of an individual older adult. The study limits itself to mapping the various factors that determine the level of functioning in a framework. It would not be correct to extrapolate the respondents’ individual perceptions to the level of functioning of the whole vulnerable older adult population.

### Implication of findings

In this study, it was the older adult’s target to perform daily routines and meaningful activities as independently as possible. In order to help older adults maintain their level of independence, healthcare professionals should consider each individual’s unique experiences. The conceptual framework presented in this paper provides elements to consider in the process of the client’s empowerment and ways to anticipate a potential lack of therapy adherence. Per phase of the TTM, elements are identified that help health professionals, among which occupational therapists, to gain insight into the older person’s perception regarding their performance and about the possible resistance and facilitators to achieve behavioral change. The occupational therapist can use this information for a client-centered approach and thus increase the chance of success of the treatment.

Future research needs to validate this conceptual framework and investigate its impact.

## Supplementary Information

**Additional file 1.** Interview guide.

**Additional file 2.** Ethical considerations.

## Data Availability

All the interviews were transcribed into text files. The text files containing the transcripts are kept in locked files, accessible only by corresponding author. The datasets generated and analyzed during the current study are not publicly available due ethical approval. The dataset is not available upon request.

## Abbreviations

ASE: Attitude-Social influence
Self: Efficacy Model
BADL: Basic Activities of Daily Living
CMOP-e: Canadian Model of Occupational Performance
GP: General Practitioner
IADL: Instrumental Activities of Daily Living
ICF: International Classification of Functioning, disability and health
OT: Occupational Therapist
TTM: TransTheoretical Model of Change
