# Supplementary material for: Perspectives of older adults with a chronic condition on functioning, social participation and health: a qualitative study
Source: BMC Geriatr. 2021 Jul 9;21:418. doi: 10.1186/s12877-021-02365-w (PMC8268461; doi:10.1186/s12877-021-02365-w)
Supplement: Supplementary file 1 — Additional file 1. Interview guide. [file 12877_2021_2365_MOESM1_ESM.docx]

Additional 1: Interview guide

**Interview guide**

*Icebreaker*

Neutral expression regarding the housing situation.

*Level of activity/participation*

Please, describe how an average day for you looks like. What types of activities do you do? What activities in the community do you participate at?

*Determining factors in general*

- What facilitates you in doing these activities/participating? (attitude, mental limitations, physical limitations, influence of environment, …)

- What stops you from doing these activities/participating? (attitude, mental limitations, physical limitations, influence of environment, …)

- How important are the influences of other people (family, friends, …) on you being active?

*Determining factor ‘Self efficacy’*

- How did activity/participating change for you over the years? What types of activity did you do/what activities did you participate in the past?

- Why don’t you do them anymore? What are conditions to do them again?

- What is your attitude to aid? Do you have support? If you need support in future, what are you thinking of?

*Demographic characteristics*

- What’s your age?

- What kind of work did you do before your retirement?

- How would you describe your general health? Do you have any health issues?

*Closure*

Do you want to add something?
