# Supplementary material for: Perspectives of older adults with a chronic condition on functioning, social participation and health: a qualitative study
Source: BMC Geriatr. 2021 Jul 9;21:418. doi: 10.1186/s12877-021-02365-w (PMC8268461; doi:10.1186/s12877-021-02365-w)
Supplement: Supplementary file 2 — Additional file 2. Ethical considerations. [file 12877_2021_2365_MOESM2_ESM.docx]

Additional 2: Ethical considerations

**Ethical consideration regarding respondents**

*Providing understandable study results to respondents:* Respondents can learn from the results of the study. They can benefit from this study by alerting them to the various factors that affect their functioning and social participation.

**Ethical considerations regarding future patients**

*Supports research that reaches a consensus on a standardized intervention protocol regarding support for functionality in daily activities and social participation:* The data from this research will be used to develop a high-quality approach for community and home-based Occupational Therapy for older adults.

*Supports research that defines client-centered goals that reflect the unique goals and preferences of the elderly:* The information gained from this research provides insight into how older adults estimate their functioning. This insight can be used to support professionals to work more consciously with and adjust their mannerism towards their clients.

**Ethical considerations regarding society**

*Provide information on efficient and effective intervention in regards to the primary care for older adults:* The results of this study will be used to define high-quality interventions for older adults. These interventions will lead to cost-effective care for home dwelling older adults and their greater satisfaction. Special attention will be paid to mapping out functioning determinants. These determinants will support the therapist in increasing adherence and client-focused work.
